# *CFTR* dysregulation drives active selection of the gut microbiome

**DOI:** 10.1371/journal.ppat.1008251

**Published:** 2020-01-21

**Authors:** Stacey M. Meeker, Kevin S. Mears, Naseer Sangwan, Mitchell J. Brittnacher, Eli J. Weiss, Piper M. Treuting, Nicholas Tolley, Christopher E. Pope, Kyle R. Hager, Anh T. Vo, Jisun Paik, Charles W. Frevert, Hillary S. Hayden, Lucas R. Hoffman, Samuel I. Miller, Adeline M. Hajjar

**Affiliations:** 1 Department of Comparative Medicine, University of Washington, Seattle, WA, United States of America; 2 Center for Microbiome and Human Health, Lerner Research Institute, Cleveland Clinic, Cleveland, OH, United States of America; 3 Department of Microbiology, University of Washington, Seattle, WA, United States of America; 4 Department Pediatrics, University of Washington, Seattle, WA, United States of America; 5 Departments of Medicine, Allergy and Infectious Disease, and Department of Genome Sciences, University of Washington, Seattle, WA, United States of America; 6 Department of Cardiovascular and Metabolic Sciences, Lerner Research Institute, Cleveland Clinic, Cleveland, OH, United States of America; Columbia University, UNITED STATES

## Abstract

Patients with cystic fibrosis (CF) have altered fecal microbiomes compared to those of healthy controls. The magnitude of this dysbiosis correlates with measures of CF gastrointestinal (GI) disease, including GI inflammation and nutrient malabsorption. However, whether this dysbiosis is caused by mutations in the CFTR gene, the underlying defect in CF, or whether CF-associated dysbiosis augments GI disease was not clear. To test the relationships between CFTR dysfunction, microbes, and intestinal health, we established a germ-free (GF) CF mouse model and demonstrated that CFTR gene mutations are sufficient to alter the GI microbiome. Furthermore, flow cytometric analysis demonstrated that colonized CF mice have increased mesenteric lymph node and spleen TH17+ cells compared with non-CF mice, suggesting that CFTR defects alter adaptive immune responses. Our findings demonstrate that CFTR mutations modulate both the host adaptive immune response and the intestinal microbiome.

## Author summary

It has been difficult to establish causal relationships between host genetics and the selection of the vast multitude of micro-organisms that live in and on us (i.e. the microbiota). Cystic fibrosis has been shown to be associated with changes in the fecal microbiome (the genetic constitution of the microbiota) although it was not evident whether mutation of CFTR, the gene mutated in CF, could drive this selection or whether the frequent use of antibiotics in this population was at fault. Here, by using a germfree (i.e. sterile, lacking all microbiota) mouse model of CF we clearly demonstrate that mutated CFTR alone can alter the microbiome. We also show an increase in an adaptive immune cell type (TH17 cells) in the mesenteric lymph nodes and spleens of CF mice compared to control mice. Our study provides new insights into the dominant role that CFTR plays in microbiome determination and suggests that therapies restoring CFTR function could also correct the microbial dysbiosis observed in CF.

## Introduction

Cystic fibrosis (CF) is a genetic disorder caused by a mutation in the cystic fibrosis transmembrane conductance regulator (CFTR) gene [1–3]. CF is the most common autosomal recessive disorder affecting Caucasians [4], currently affecting >30,000 patients in the US and >70,000 patients worldwide [4, 5] with approximately 1000 new cases diagnosed each year [5, 6]. The CFTR gene encodes a cAMP-regulated anion channel [7, 8] and mutations in the CFTR gene lead to ion imbalance and dysregulation of fluid secretions within a variety of organs including lung, GI and reproductive tracts, pancreas, and skin [6, 9].

More than 2000 mutations of the CFTR gene have been identified that cause disease of variable severity [6, 10]. While respiratory disease is a widely recognized source of morbidity, CF patients also exhibit an array of GI symptoms. At birth, infants with CF can develop meconium ileus, an intestinal obstruction that often has to be treated surgically. Distal intestinal obstructive syndrome also occurs in older individuals. In addition, patients usually exhibit destruction of the pancreas that begins *in utero* and progresses throughout life, leading to pancreatic exocrine insufficiency that requires lifelong pancreatic enzyme replacement therapy [11]. All of these manifestations of disease are likely the result of the altered mucosal secretions and physicochemical conditions that CFTR dysfunction produces [12–14].

Observational studies have identified a fecal dysbiosis in infants and children with CF compared with those without CF. The childhood CF fecal dysbiosis is characterized by increased relative abundances of Proteobacteria, especially *E*. *coli*, and decreased Clostridiales, compared to infants without CF [15–17]. The magnitude of this dysbiosis correlates with a fecal measure of intestinal inflammation, calprotectin, and also with fecal fat, which is malabsorbed due to pancreatic insufficiency. However, while the CF GI dysbiosis could be caused by either inflammation or pancreatic exocrine dysfunction, the GI microbiome could also contribute to these problems. Therefore, whether and how the CF GI dysbiosis contributes to disease progression, and the causes of the dysbiosis itself, are unclear [15, 18]. Furthermore, people with CF consume more high-energy and high-fat diets compared to controls [19] and are frequently prescribed antibiotics and antifungal drugs to treat their lung infections, therapies that likely further disturb the gut microbiome in these patients. Additionally, as patients with CF live longer, they are at increased risk of developing cancers in the gastrointestinal tract, diseases with known relationships with the GI microbiome and inflammation outside of CF [20–22].

The gut microbiome is now known to influence the development and severity of many diseases within and distant to the GI tract. Given the evidence in CF, we sought to dissect the role of the microbiome in CF gastrointestinal disease. To accomplish this goal, we generated germ-free (GF) CF mice to eliminate the role of microbiota and to determine what phenotypes could be attributed solely to CFTR dysfunction. To our knowledge, this is the first report of the establishment and characterization of a GF CF mouse model.

## Results

### Establishment of a germ-free CF mouse model

**Fig 1:**
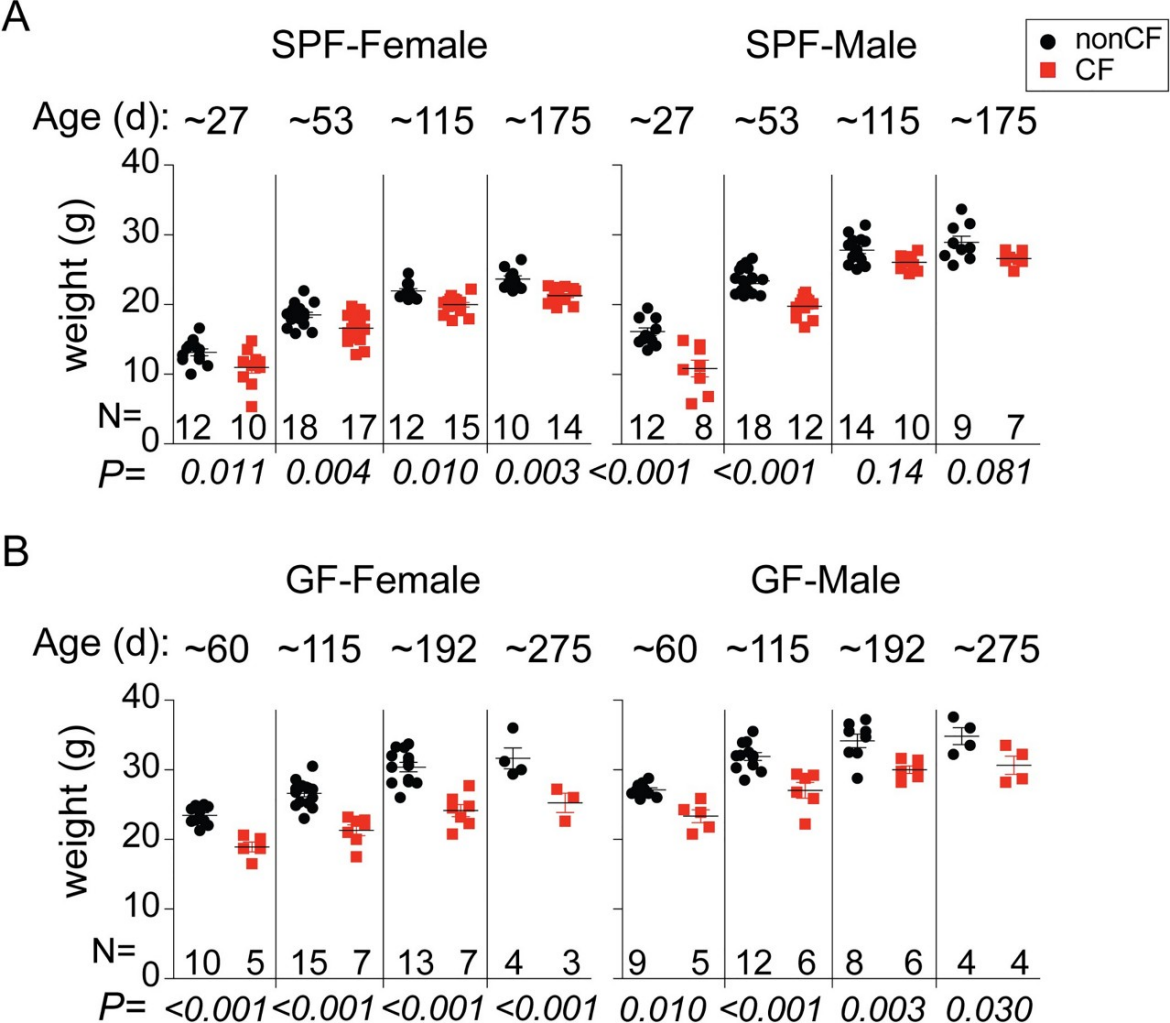
CF mice have significantly lower body weights compared to age matched non-CF mice. Body weight was measured at multiple ages in male and female SPF **(A)** and GF **(B**) mice. Number of mice per group (N) is indicated along the X axis for each time point. Body weight (grams) is plotted for each animal and mean weight and SEM for each group is indicated. Data were analyzed by two-way ANOVA with the Bonferroni correction for multiple comparisons (*P* values).

To determine the contribution of the microbiota to CF disease phenotypes, we established a GF CF mouse model at the University of Washington Gnotobiotic Animal Core (GNAC). The GF CF mouse model was generated and maintained through breeding of heterozygous male and female mice. Similar to what has been previously reported [23], we found a lower than expected proportion of CFTR knockout (CF) mice that were successfully weaned from heterozygote mating for both GF and specific pathogen free (SPF) mice (Table 1). Because CFTR mutations have been associated with stunted growth in both humans and in animal models [18, 24–27], we evaluated the growth rates of both GF and SPF CF and non-CF mice over time. Consistent with previous findings [23], we found that both male and female SPF CF mice had significantly lower body weights compared to non-CF controls from weaning through ~8 weeks of age, although this phenotype was lost with age in male SPF mice but not female mice (Fig 1A). Similarly, GF male and female CF mice had significantly lower body weights compared to age matched GF non-CF mice for the duration of the study (Fig 1B).

**Table 1:** Proportion of CF animals produced.

	SPF	GF
Total Number of Litters Evaluated	123	124
Total Number of Pups Weaned	562	496
Number of CFTR wildtype (% of total)	169 (30.1%)	127 (25.6%)
Males / female	81 / 88	68 / 59
Number of CFTR heterozygous (% of total)	329 (58.5%)	304 (61.3%)
Males / female	171 / 158	159 / 145
Number of CFTR knockouts (% of total)	64 (11.4%)	64 (12.9%)
Males / female	30 / 34	33 / 31

### GF CF mice demonstrate similar histopathologic findings compared to SPF CF mice

**Fig 2:**
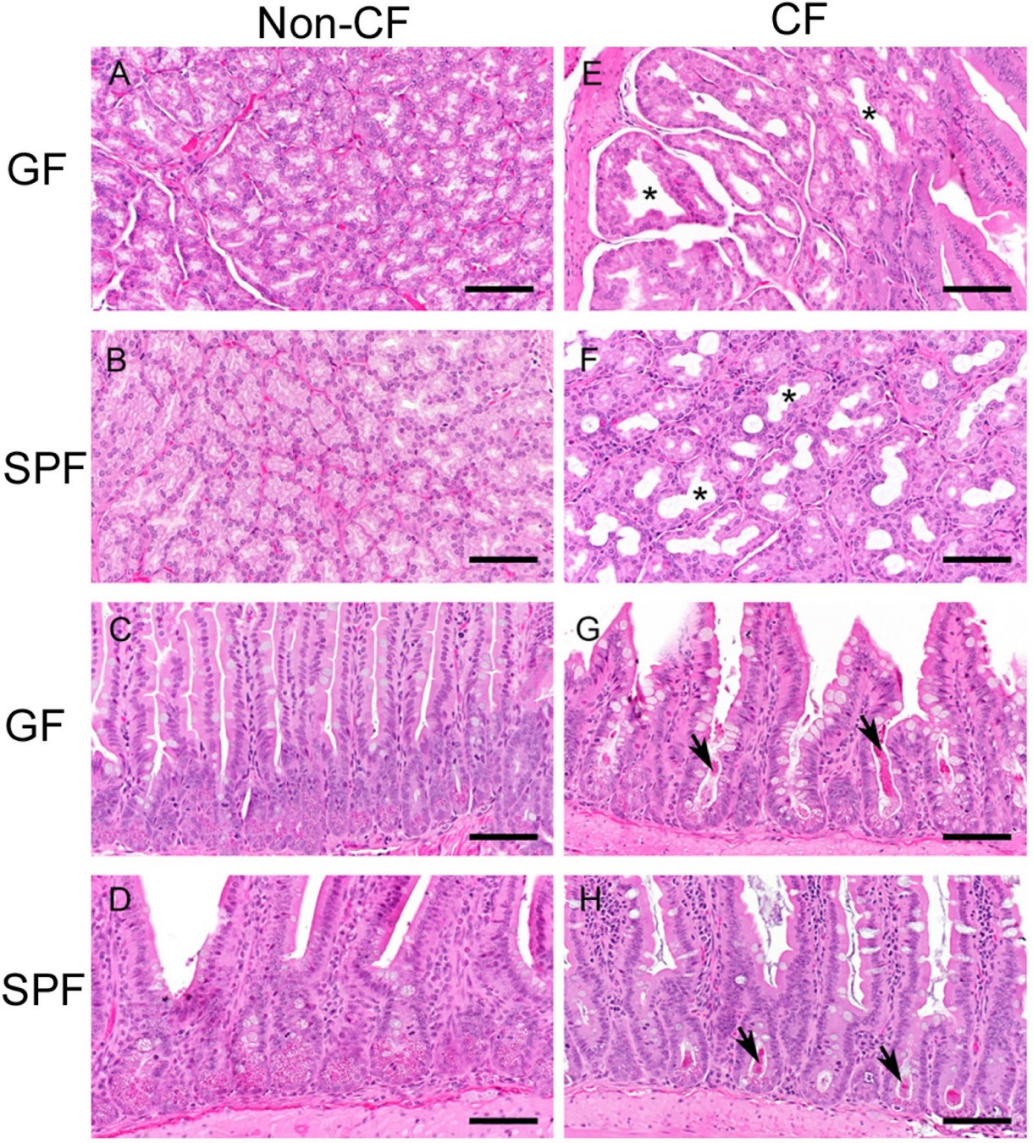
GF CF mice recapitulate common histologic findings observed in SPF CF mice. Representative H&E stained sections from non-CF **(A-D)** and CF **(E-H)** mice. Microbiological status as indicated, germ-free (GF); specific pathogen free (SPF). **(A, B & E, F)**- Brunner’s glands. **(C,D & G, H)**- Small intestine. The non-CF tissues are within normal or anticipated limits for SPF-colonized (minimal to mild enteritis) or GF mice. In contrast, the CF mice have dilated Brunner’s glands [* in **(E and F)**] and retention of Paneth cell contents on the deep intestinal crypts [arrows in **(G and H)**]. These changes are present in SPF and GF CF mice to equal measure. SPF animals of both genotypes have mild proliferative enteritis as compared to GF animals. Scale bars~100um.

In order to further characterize the GF CF mouse model, we performed gross necropsies on GF CF and GF non-CF mice as well as SPF CF and non-CF mice. Tissues were evaluated histologically by a board-certified veterinary pathologist masked to genotype. No significant lesions were appreciated on gross examination in any of the mice. All GF mice, regardless of genotype, had markedly dilated ceca with liquid ingesta which is a well characterized finding in GF mice [28], and could account in part for differences observed in body weight between GF and SPF animals (Fig 1A and 1B). The histologic findings in GF CF mice were consistent with those that we observed in SPF mice and that have been previously reported in this model [23, 29]. Compared with non-CF mice (Fig 2A–2D), both GF and SPF CF mice demonstrated significant dilation of the Brunner’s glands within the proximal small intestine (Fig 2E and 2F) and retention of Paneth cell contents in the deep intestinal crypts (Fig 2G and 2H). In addition, mucus retention within the goblet cells was apparent throughout the intestinal tract. SPF CF mice had protozoal blooms within the luminal contents of the cecum that were not observed in non-CF controls. Similar to previous reports, we did not observe consistent lesions within the lung or pancreas of GF or SPF CF mice.

### CF genotype significantly influences the gut microbiota

**Fig 3:**
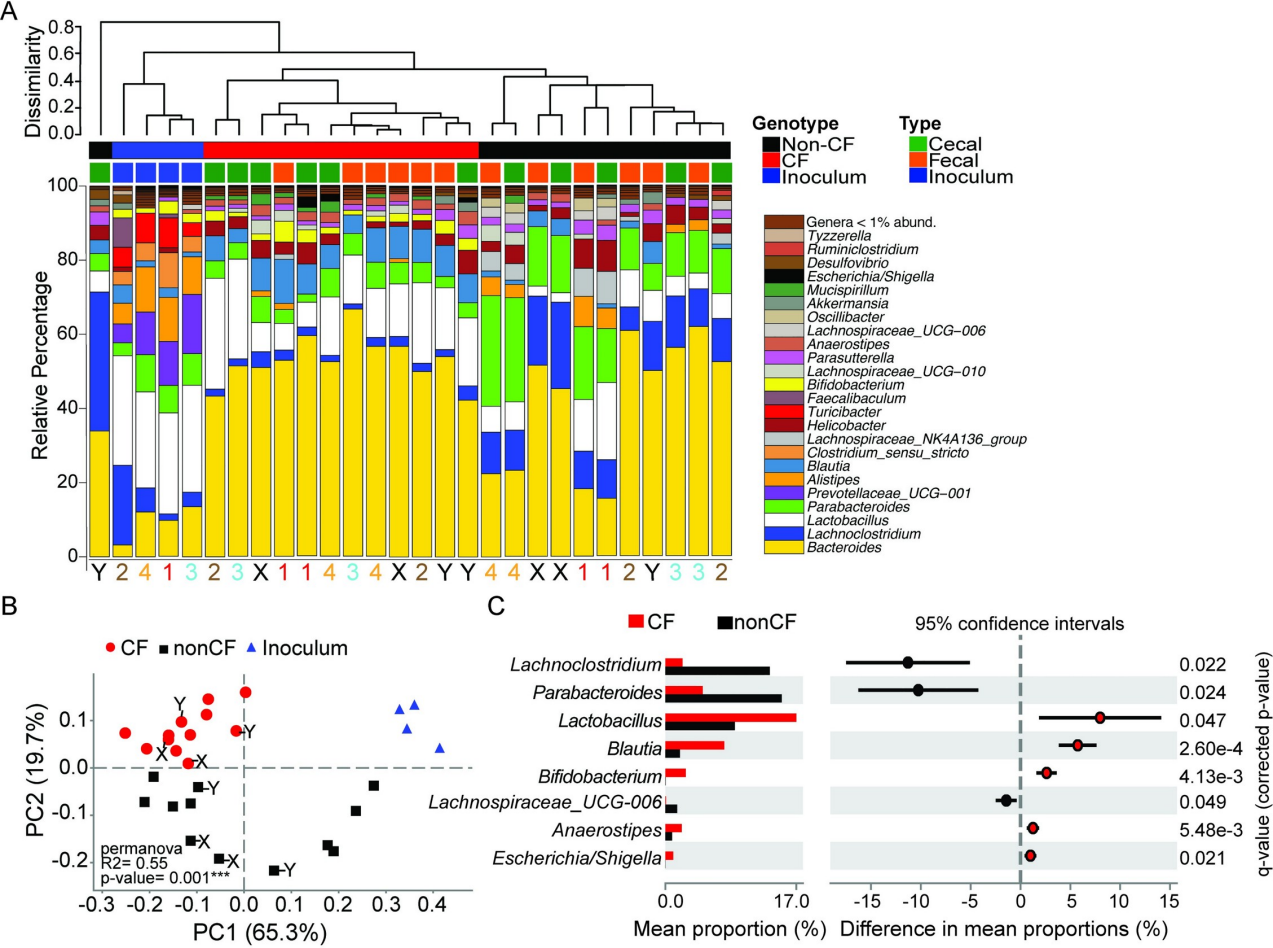
Mutations in CFTR gene influence the composition of the gut microbiota. The composition of the fecal and cecal microbiota were assessed in CF and non-CF GF recipient mice 1–3 months following FMT with SPF C57BL/6J feces by sequencing of the V3-V4 hypervariable regions of the 16S rRNA gene. See also S1 Fig. **(A)** Bar graph represents the relative abundances of major genera identified within each sample. Hierarchical clustering was performed based on Bray-Curtis distance, according to ASV distribution at the genus level. The experiment was repeated four times over the course of a year and the numbers (and colors) at the bottom of each column (Red, Brown, Blue, Orange) correspond to an experimental cohort. Fecal and cecal contents were pooled at equal volumes for pair-housed, age, sex, and genotype matched mice within a study prior to DNA extraction (N = 2 mice per genotype per experiment). Black X and Y samples indicate samples from two experiments that used the equivalent of the CF cecal sample of experiment 4 as donor material for the FMT. **(B)** Multidimensional scaling by ASV’s abundance demonstrate significant clustering by genotype (PERMANOVA, p <0.05, with R2 = 0.55). Red circles represent CF whereas black squares represent non-CF samples. Blue triangles are inoculum samples of SPF C57BL6/J mice bred inhouse. **(C)** Genera significantly enriched or depleted in CF mice and associated q-values from experiments 1–4 (i.e. excluding the X and Y samples) as determined by White’s non-parametric t-test; CI method: Welch’s inverted; multiple test correction method: Benjamini & Hochberg.

Once the GF CF mouse model was established, we sought to determine the influence of the host CFTR mutation on shaping the gut microbiome. To accomplish this goal, we performed a series of fecal microbiota transplant (FMT) experiments in which GF CF and non-CF mice were housed separately by genotype and colonized with fecal inoculum from SPF C57BL/6J mice. After allowing sufficient time for the microbiome to stabilize following the FMT [28], we examined the microbial composition of the feces and cecal material of CF and non-CF animals by 16S rRNA gene sequencing. Alpha diversity was greatest in the inoculum and was reduced in both fecal and cecal samples collected from CF compared to non-CF mice (S1 Fig). Hierarchical clustering of samples by their relative species abundance revealed a clear separation based on CFTR genotype (Fig 3A) demonstrating that CFTR dysfunction alone is sufficient to modulate the gut microbiome. Principal coordinate analysis (PCoA) of the community composition similarly indicated that the samples significantly (PERMANOVA, p <0.05) diverged based on CFTR genotype (Fig 3B). Furthermore, samples diverged from the starting inoculum with non-CF recipients showing clustering of cecal content and fecal content within a given experiment for 3 of the 4 experiments unlike CF recipients that overall seemed to cluster more closely together (Fig 3A and 3B). Surprisingly, when CF cecal content was used as the donor sample, the microbiome reverted to non-CF-like in the non-CF mice (recipients marked with X or Y in Fig 3A and Fig 3B), further suggesting the important role of CFTR function in GI microbiota composition. Genera that showed significant (White’s non-parametric t-test, p < 0.05) enrichment or depletion in CF compared to non-CF mice are shown in Fig 3C. Strikingly, we saw a significant enrichment in the proportion of *Escherichia/Shigella* (~250-fold, though only at a low abundance of 1.01 ± 0.78% in CF compared to 0.004 ± 0.012% in non-CF mice). In addition, we also saw a depletion of *Lachnoclostridium* (2.22 ± 0.66% in CF compared to 13.5 ± 8.2% in non-CF mice) and *Parabacteroides* (4.83 ± 1.62 in CF compared to 15.1 ± 7.9% in non-CF). These results are consistent with observations in pediatric human CF fecal microbiome studies [15, 17, 30].

### CF genotype influences the TH17 immune response in mice

**Fig 4:**
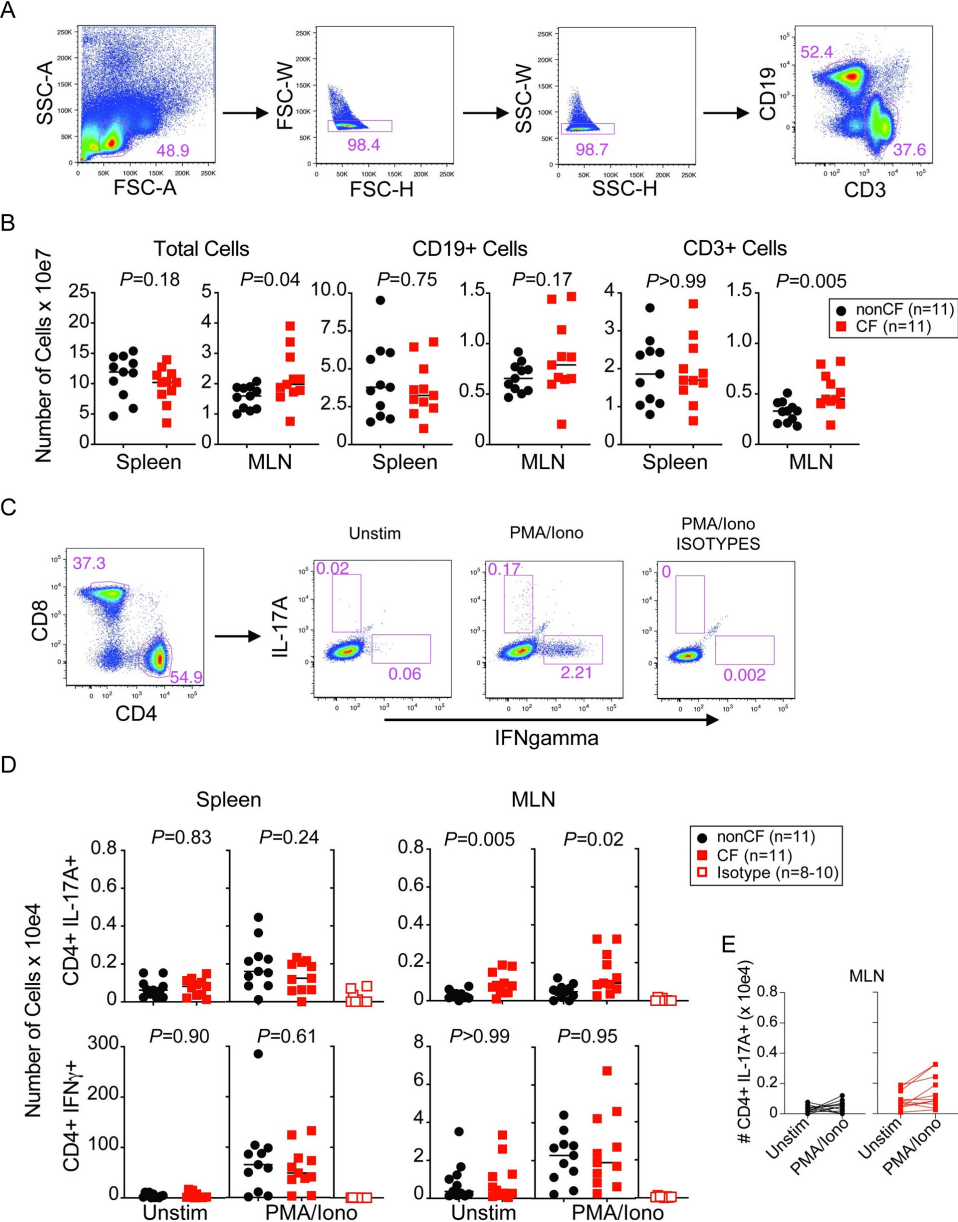
GF CF mice exhibit elevated TH17 cells in the MLN compared to non-CF controls. **(A)** Gating strategy used to enumerate cell numbers **(B)** of T (CD3+) and B (CD19+) cells in the Spleen and MLN from GF CF and non-CF mice (N = 11 mice per genotype). **(C)** Gating strategy to identify IFNgamma+ or IL-17A+ CD4+ T-cells following ~5 hours of stimulation in the presence of PMA / Ionomycin. Note that the gating strategy in this figure (including Panel **(A)**) comes from an FMT mouse to show IL-17A production. **(D)** Cell counts per mouse (N = 11 mice per genotype, and N = 8 MLN and N = 10 Spleen samples that were also stained with isotype control antibodies in parallel). **(E)** Data from **(D)** but showing IL-17A production linking Unstim to PMA/Ionomycin stimulation for each mouse. Data were analyzed by pairwise comparison between CF and non-CF animals within a group by Mann-Whitney U test.

The host immune system plays an important role in shaping the gut microbiome, and conversely the gut microbiome can significantly modulate the host immune system. The literature suggests that CF patients with a TH17 biased T cell immune response may have poorer disease outcomes [31, 32]. We therefore sought to determine if there were any changes in the adaptive immune cell populations between CF and non-CF mice prior to and following colonization. We first evaluated total cellularity of the spleen and mesenteric lymph nodes (MLN) of unmanipulated GF CF and non-CF mice using flow cytometric analysis (Fig 4). CF mice demonstrated a higher total cell count as well as a higher number of CD3+ T cells in the MLN compared to non-CF controls, although no significant differences were observed in the spleen (Fig 4B). Additionally, no difference in total number of B cells (CD19+) was observed in the MLN or the spleen (Fig 4B). To further assess T helper cell differentiation, we stimulated whole splenocytes or MLN with phorbol 12-myristate 13-acetate (PMA) and ionomycin and stained for intracellular cytokine production in CD4+ T-cells (Fig 4C). We found an increase in TH17 cells in the MLN in the CF mice compared to the non-CF controls following PMA / ionomycin stimulation in 4 of 11 mice tested (Fig 4E). In addition, the MLN already showed an increase in intracellular IL-17A, the cytokine produced by TH17 cells, even in the absence of stimulation, although the number of cells was low (median of 7.8 x 10e2 in CF vs 2.1 x 10e2 in non-CF). No significant differences in TH17+ cells were observed in the spleen. Additionally, there were no differences in TH1 cells in the MLN or spleen (Fig 4D).

**Fig 5:**
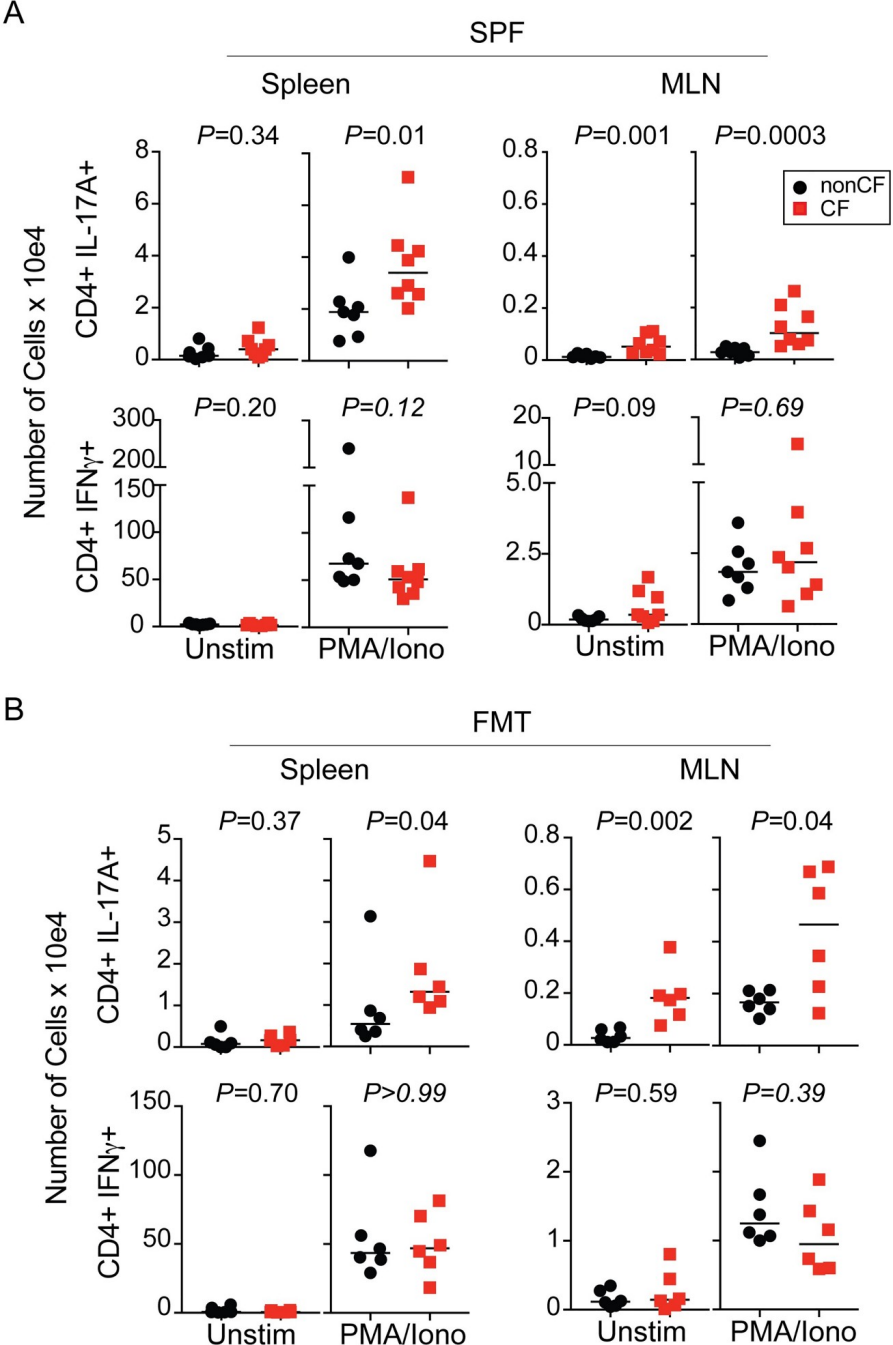
SPF and FMT-colonized CF mice exhibit elevated TH17 cells in the spleen and MLN compared to non-CF controls. Number of CD4+ T-cells producing IFNgamma or IL-17A in the spleen or MLN of SPF [(**A)** N = 7–8 mice per genotype] or B6-FMT [(**B)** N = 6 mice per genotype, not performed in 1^st^ FMT experiment] mice. Data were analyzed by pairwise comparison between CF and non-CF animals within a group by Mann-Whitney U test. See also S2 Fig.

We next sought to determine if the increased TH17 response observed in the GF CF mice was altered by the presence of a diverse GI microbiome. We evaluated the adaptive immune cell populations between CF and non-CF mice in our SPF colony (Fig 5A) and following colonization after FMT (Fig 5B). Similar to what was observed in the GF CF mice, SPF CF mice had a significantly higher total cell count and a larger number of T cells in the MLN compared to non-CF mice, whereas CF and non-CF FMT mice had equivalent counts (S2 Fig). SPF CF mice also had increased B cells in the MLN, but there were no significant differences in the number of T or B cells present in the spleen (S2 Fig). Intriguingly, the SPF and FMT-colonized CF animals exhibited increased TH17 cell populations compared to non-CF controls in both the spleen and the MLN (Fig 5) with no differences observed in the number of TH1 cells present. Together these data demonstrate that CFTR dysregulation modulates the host immune system, leading to an increase in TH17 cells, an immune phenotype associated with worse outcomes in CF.

## Discussion

Both pediatric and adult CF patients have a significantly altered fecal microbiome compared to healthy controls, and it has been suggested that GI dysbiosis may be associated with increased morbidity in CF patients. However, it is difficult within a human population to determine both the causes and consequences of the CF fecal dysbiosis for clinical disease due to the large number of confounding variables such as antibiotic use, diet, and other underlying genetic and environmental influences that are present within a diverse human population. Gnotobiotic animal models provide an opportunity to directly evaluate the effects of the gut microbiome on health and disease. We therefore sought to establish a gnotobiotic mouse model of CF that could be used to dissect the complex interactions between *CFTR* dysregulation and the gut microbiome. We rederived and established a breeding colony of CFTR S489X mice. Using these animals, we found that GF CF mice demonstrated histopathologic changes within the GI tract similar to those previously reported in SPF CF animals, including dilatation of the Brunner’s glands in the proximal small intestine, retention of mucus within the intestinal goblet cells and retention of the Paneth cell contents within the intestinal crypts. While the intestinal histopathologic changes associated with CFTR dysregulation are understudied, inspissated eosinophilic mucus within the goblet cells and intestinal crypts is a characteristic finding in human CF patients as well [33, 34]. Intriguingly, we found that, similar to SPF animals, GF CF mice had decreased body weights compared to age and sex matched non-CF controls even when maintained on a nutritionally dense diet. Inadequate growth is common in human CF patients as well as multiple animal models of CF [18, 24–27]. Our findings suggest that reduced body weight in mice with CF is not driven by the presence of gut microbes.

By using the same donor microbiota to colonize CF and non-CF mice under identical housing conditions, our results demonstrate that CFTR dysfunction actively modulates the gut microbiome and selects for a community with features that are conserved between mice and humans. We found an increase in *Escherichia*, though at a lower abundance than that observed in human infants, where *E*. *coli* exhibits unique traits to grow in the CF intestinal environment [16]. In addition, as in the children studied by Manor *et al*. [17], we found an increase in the relative abundance of *Bifidobacterium* in CF mouse samples. In contrast, while human CF samples exhibited lower overall relative abundances of Firmicutes, only two Firmicute genera (*Lachnoclostridium* and *Lachnospiraceae*_UCG-006) exhibited a significant depletion in CF versus non-CF samples (the other three Firmicutes found in murine GI microbiota, *Lactobacillus*, *Blautia*, and *Anaerostipes*, were enriched in CF samples). These differences may be attributable to specific features of the murine GI lumen, or perhaps to the specific diets and therapies that children with CF receive, which are therefore important confounders in human studies [17].

As further evidence of the importance of CFTR function as a selective force in the mouse GI lumen, we showed that when non-CF mice are colonized with CF-selected microbiota, they revert to a non-CF microbiota. There are multiple potential mechanisms by which CFTR dysregulation could modulate the gut microbiome. First, the nutrient availability impacted by CFTR function, such as altered mucus abundance or composition, or malabsorbed dietary nutrients [15, 17], could contribute to this selection. Second, altered pH due to decreased bicarbonate secretion could directly (through direct effects on microbiota) or indirectly (by impacting the activity of another factor, such as antimicrobial peptides) alter microbiota composition. Third, Paneth cells secrete a variety of MyD88-dependent antimicrobial compounds that protect the epithelium [35–37], and trapping of the Paneth cell granules in the crypts could alter their antimicrobial activity in the CF gut. Future studies will be required to differentiate between these mechanisms.

Finally, we observed increased TH17 responses in the spleen and MLN of CF mice upon GI microbial colonization. Increased intestinal permeability has been demonstrated in human CF [38, 39] and could account for increased bacteremia leading to increased systemic TH17 cells. Alternatively, differences in specific taxa such as increased *Escherichia*, could be the driver of these immune responses, though these hypotheses will require further testing. It has been reported that CF patients exhibit a skewed T cell mediated immune response with a bias towards a TH2 or TH17 response [40–42]. In addition, increased TH17 responses were associated with decreased lung function [31]. Interestingly, Kushwah *et al*. demonstrated that naïve T cells isolated from peripheral blood of human CF patients exhibit TH17-skewed responses [40]. However, these studies could not establish whether this skewed inflammatory response was secondary to environmental antigen exposures or a direct result of CFTR dysfunction. Our results suggest that TH17-skewed T-cell differentiation in CF is not entirely dependent upon the composition of the gut microbiome, as we found a small number of IL-17A-producing CD4+ T-cells in the MLN of a subset of GF CF mice. While our data are intriguing, additional studies are required to determine whether these cells are indeed TH17 cells and what factors are driving their differentiation in the absence of gut bacteria, such as exposure to environmental or food antigens, or T-cell intrinsic factors. Together, our data demonstrate that CFTR dysfunction modulates the adaptive immune response and the gut microbiome suggesting that restoring CFTR function through CFTR modulators could result in the added benefit of the reversion of the gut microbiota towards that found in healthy, non-CF people, and such findings were recently described in a small prospective observational study where ivacaftor treatment resulted in altered gut microbiota and decreased intestinal inflammation [43].

## Materials and methods

### Animals

Male and female CFTR S489X (B6.*Cftr*^*tm1Unc/+*^)[23] mice were obtained from Dr. Mitchell Drumm at Case Western Reserve University and bred at the University of Washington to generate homozygous mutant (CF), heterozygous and wildtype mice. A combination of heterozygous and wildtype animals were used as non-CF experimental controls throughout this manuscript. C56BL/6J mice, originally obtained from Jackson Laboratories, were bred and maintained at the University of Washington. Specific pathogen free (SPF) mice were group housed (2–5 mice per cage) in autoclaved, individually ventilated cages (Thoren, Hazleton, PA or Allentown, Allentown, NJ) with autoclaved corncob bedding (The Andersons, Maumee, OH). Mice were fed an irradiated chow diet (Rodent Diet 5053, Lab Diet, St. Louis, MO for C57BL/6J mice and Rodent Diet 7960, Harlan Teklad, Madison, WI for CF mice) ad libitum, provided acidified (pH 2.5–3), autoclaved, reverse osmosis purified water in bottles, and maintained in rooms with a 12/12 or 14/10 hour light / dark cycle. Mice were screened for rodent pathogens by either monitoring of sentinel mice (Crl:CD1[ICR]; Charles River, Wilmington, MA) or exhaust air duct PCR testing to ensure exclusion of the following agents: mouse hepatitis virus, mouse parvovirus, epizootic diarrhea of infant mice virus, minute virus of mice, pneumonia virus of mice, Reovirus type 3, Sendai virus, Theiler’s murine encephalomyelitis virus, lymphocytic choriomeningitis virus, Ectromelia virus, Mycoplasma pulmonis, pinworms and fur mites.

In order to assess the role of the microbiome in the CFTR intestinal phenotype, CFTR S489X mice were rederived germ-free (GF) via caesarian section in the Gnotobiotic Animal Core (GNAC) Facility at the University of Washington. A breeding colony of GF CF mice was maintained by breeding heterozygous carriers in a flexible film isolator (CBC, Madison, WI) in open top cages. Experimental mice were transferred into ISOcage P cages (Tecniplast, West Chester, PA) prior to colonization, and the GF status of animals was determined by 16S rRNA PCR on pooled cage feces [28]. Handling of mice in ISOcage P cages was performed in a biosafety cabinet as previously described [28]. Gnotobiotic mice were all housed within rooms in the GNAC facility at the University of Washington with a 12/12 or 14/10 hour light / dark cycle. Mice were housed on Enrich-n’Pure bedding (The Andersons), fed autoclaved chow diet (Rodent Diet 5021, Lab Diet, St. Louis, MO) ad libitum, and provided reverse-osmosis purified autoclaved water in bottles.

All experimental animals, regardless of genotype or colonization status were maintained on laxative water upon weaning. Laxative solution consisted of 50% Gavilyte-C solution prepared in animal’s respective colony water. Body weight was assessed weekly in experimental animals.

### Ethics statement

All animal procedures were approved by the Institutional Animal Care and Use Committee of the University of Washington (UW protocols 4230–01 and 4420–02) in accordance with the recommendations in the Guide for the Care and use of Laboratory Animals, 8^th^ Edition, by the National Research Council of the National Academies (US). The UW Public Health Service Assurance Number by the Office of Laboratory Animal Welfare is D16-00292 (A3464-01).

### Fecal microbiota transplantation (FMT)

Fresh fecal pellets from five cages of five SPF C57BL/6J mice were collected over the course of five days and frozen at -80°C. Each tube consisted of a single fecal pellet from one mouse from each of the five cages thus containing a total of five pellets. Frozen fecal pellets were resuspended in PBS at 80–100 mg/mL, centrifuged at 27–100 rcf for 10 seconds, and 0.1 mL of the supernatant was administered to each GF mouse by oral gavage at day 0, when the recipients were 5–8 weeks of age. This experiment was repeated 4 times over the course of one year with each experiment consisting of a cage of two CF mice and a cage of two non-CF mice. However, one CF mouse was found dead 8 days after gavage in the first experiment. This animal was excluded from study and therefore the total analyzed was 7 KO (4F/3M), 4WT (2M/2F), and 4 hets (2M/2F). The 1^st^ 3 experiments extended to 3 months whereas the last one extended to 28 days. In addition, intracellular cytokine staining was only performed on the last 3 of the experiments.

### Tissue collection and histopathology

Mice were euthanized via CO_2_ asphyxiation or cervical dislocation. Spleen and mesenteric lymph node (MLN) were collected for flow cytometric analysis from a subset of animals. Tissues including spleen, liver, kidney, heart, lung, stomach, cecum, MLN with mesentery and pancreas, colon and small intestine were immersion fixed in 10% neutral-buffered formalin, embedded in paraffin, sectioned, and stained with hematoxylin and eosin. Tissues were evaluated by a board-certified veterinary pathologist (PMT), masked to experimental genotype or treatment.

### Flow cytometric analysis

Single cell suspensions of splenocytes were obtained by mechanical disruption of the spleen between frosted glass slides in RBC-lysis solution followed by washing in medium (RPMI + Glutamax + beta mercapto ethanol + 10% FBS + Pen/Strep). MLN were dissociated in medium by passing through a 40 μm filter using the plunger of a syringe. Cells were then cultured for ~5 hr in the presence of 10 μg/mL brefeldin A (Sigma-Aldrich, St. Louis, MO) in medium and stimulated with 10 ng/mL mL Phorbol 12-myristate 13-acetate (Sigma-Aldrich) plus 2.8 μM Ionomycin (Sigma-Aldrich). Using a hemocytometer, live cells were counted (trypan blue exclusion) and a total cell count was obtained for each sample. Surface cell Fc receptors were blocked with 2.4G2 antibody then cells were stained with combinations of fluorescently labeled antibodies specific for (mouse) CD3, CD19, CD4, and CD8 (BD Biosciences, San Jose, CA; eBioscience, San Diego, CA; or Biolegend, San Diego, CA). Cells were washed and permeabilized with Cytofix/Cytoperm (BD Biosciences) according to the manufacturer’s instructions prior to intracellular cytokine staining using fluorescently labeled antibodies specific for (mouse) IL-17A and IFN-gamma (BD Biosciences or Biolegend). Data were collected on an LSRII cytometer (BD Bioscience) and analyzed using FlowJo version 9.9.6 software (FlowJo LLC, Ashland OR). Percent of T and B cells were determined from a lymphocyte-size gate based on FSC-A vs. SSC-A and single cells determined by FSC-H vs FSC-W and SSC-H vs SSC-W.

### 16S rRNA gene sequencing and analysis

Fecal and cecal contents were pooled at equal volumes for co-housed, age, sex, and genotype matched mice within a study prior to DNA extraction. Genomic DNA was extracted from mouse fecal and intestinal contents using ZR Fecal DNA Microprep kit (Zymo Research, Irvine, CA) following manufacturer’s protocol. Final DNA yield was quantified using a Qubit fluorometer (ThermoFisher Scientific, Waltham, MA) before next generation sequencing library construction.

16S rRNA gene amplicon libraries were prepared according to Illumina’s 16S metagenomics sequencing library protocol (Illumina Inc., San Diego, CA). Briefly, an approximate 460 bp region within the V3-V4 region of the 16S rRNA gene was amplified by PCR using the forward primer S-D-Bact-0341-b-S-17 and reverse primer S-D-Bact-0785-a-A-21 [44] with Illumina 5’ overhang adapter sequences: Forward: 5’-TCGTCGGCAGCGTCAGATGTGTATAAGAGACAGCCTACGGGNGGCWGCAG-3’ and Reverse: 5’-GTCTCGTGGGCTCGGAGATGTGTATAAGAGACAGGACTACHVGGGTATCTAATCC-3’. Each sample was amplified in triplicate, pooled, and gel purified using the QIAquick Gel Extraction Kit (Qiagen, Germantown, MD). The PCR amplicon was purified using Agencourt AMPure XP beads (Beckman Coulter Genomics, Danvers, MA). Dual indices and Illumina sequencing adapters were attached to the amplicon in a limited cycle PCR reaction using the Nextera XT Index Kit (Illumina Inc.). Libraries were again purified using Agencourt AMPure XP beads. Final libraries were quantified, pooled and denatured for sequencing on the Illumina MiSeq platform. The MiSeq run generated an average of 220,000 paired 300 base pair reads per sample.

### Microbiome analysis

Illumina reads were first trimmed using Cutadapt [45] to remove all primer and adapter sequences. The resulting reads were then truncated to 260 bases for the forward read and 220 bases for the reverse read based on the observed quality scores. The DADA2 pipeline [46] was then used to infer amplicon sequence variants (ASVs). Taxonomy was assigned to the ASVs up to the genus level using the DADA2’s pre-compiled silva_nr_v128_train_set database. Community composition and statistical analyses were performed using the vegan (CRAN, Community Ecology Package), microbiomeSeq (https://github.com/umerijaz/microbiomeSeq), and Phyloseq [47] R packages. Raw and analyzed data were deposited in the European Nucleotide Archive (accession number PRJEB32648).

### Statistics

Statistical analyses for bodyweight, flow cytometric, and cell count data were performed using Prism version 5 and 7 (GraphPad, San Diego, CA). For pairwise comparisons for flow cytometric data and cell count data, the Mann-Whitney U test was used. Body weight was assessed by a two-way ANOVA with Bonferroni’s multiple comparisons test to compare body weight at each time point between sex and colonization status (GF versus SPF). Statistical analysis of the gut microbial community was performed in R (R Core Team) and STAMP [48]. Multigroup beta-dispersion analysis was done by Permutational Analysis of Variance (PERMANOVA, Benjamini-Hochberg) to compare categorical and continuous variables using microbiomeSeq (https://github.com/umerijaz/microbiomeSeq) software package in R. A two-sided White’s non-parametric t-test (CI method: Welch’s inverted) was conducted to identify statistically different Genera and ASV’s with a Benjamini & Hochberg [49] correction for multiple testing. Differences with a mean proportion of 0.2 and P value of 0.05 or less were considered significant.

## Supporting information

S1 Fig Reduced alpha-diversity in CF mice. Alpha-diversity, measured by Simpson and Shannon diversity indexes is plotted for fecal and cecal microbiome samples assessed in CF (red) and non-CF (green) germ-free recipient mice 1–3 months following FMT with SPF C57BL/6J feces as the inoculum (blue). Fecal and cecal contents were pooled at equal volumes for pair-housed, age, sex, and genotype matched mice within a study prior to DNA extraction (N = 2 mice per genotype per experiment, four separate experiments performed).The line inside the box represents the median, while the whiskers represent the lowest and highest values within the 1.5 interquartile range (IQR). Pair-wise ANOVA of diversity measures was performed between groups and values for each of the selected methods (observed values) were plotted (annotated with significance labels:*p<0.05; **p<0.01; ***p<0.001).
(TIF)

S2 Fig SPF and colonized CF mice exhibit increased total cell counts as well as increased numbers of B and T cells in the MLN. Total cell counts in addition to number of B cells (CD19+) and T cells (CD3+) present in Spleen and MLN from CF and non-CF mice in SPF [(**A**) N = 7–8 mice per genotype] or B6-FMT mice [(**B**) N = 6 mice per genotype, not performed in 1^st^ FMT experiment]. Data were analyzed by pairwise comparison between CF and non-CF animals within a group by Mann-Whitney U test.
(TIF)

S1 Table Flow cytometry antibodies and reagents. (DOCX)

## References

[1] Riordan JR, Rommens JM, Kerem B, Alon N, Rozmahel R, Grzelczak Z, et al Identification of the cystic fibrosis gene: cloning and characterization of complementary DNA. Science. 1989;245(4922):1066–73. 10.1126/science.2475911 . 2475911

[2] Kerem B, Rommens JM, Buchanan JA, Markiewicz D, Cox TK, Chakravarti A, et al Identification of the cystic fibrosis gene: genetic analysis. Science. 1989;245(4922):1073–80. Epub 1989/09/08. 10.1126/science.2570460 . 2570460

[3] Rommens JM, Iannuzzi MC, Kerem B, Drumm ML, Melmer G, Dean M, et al Identification of the cystic fibrosis gene: chromosome walking and jumping. Science. 1989;245(4922):1059–65. Epub 1989/09/08. 10.1126/science.2772657 . 2772657

[4] Jackson AD, Goss CH. Epidemiology of CF: How registries can be used to advance our understanding of the CF population. J Cyst Fibros. 2018;17(3):297–305. Epub 2017/12/26. 10.1016/j.jcf.2017.11.013 . 29275954

[5] Cystic Fibrosis Foundation. Cystic Fibrosis Foundation Patient Registry 2017 Annual Data Report. https://www.cff.org/Research/Researcher-Resources/Patient-Registry/2017-Patient-Registry-Annual-Data-Report.pdf

[6] Ratjen F, Bell SC, Rowe SM, Goss CH, Quittner AL, Bush A. Cystic fibrosis. Nat Rev Dis Primers. 2015;1:15010 Epub 2015/01/01. 10.1038/nrdp.2015.10 . 27189798 PMC7041544

[7] Welsh MJ, Smith AE. Molecular mechanisms of CFTR chloride channel dysfunction in cystic fibrosis. Cell. 1993;73(7):1251–4. Epub 1993/07/02. 10.1016/0092-8674(93)90353-r . 7686820

[8] Csanady L, Vergani P, Gadsby DC. Structure, Gating, and Regulation of the Cftr Anion Channel. Physiol Rev. 2019;99(1):707–38. Epub 2018/12/06. 10.1152/physrev.00007.2018 . 30516439

[9] Rey MM, Bonk MP, Hadjiliadis D. Cystic Fibrosis: Emerging Understanding and Therapies. Annu Rev Med. 2019;70:197–210. Epub 2018/10/13. 10.1146/annurev-med-112717-094536 . 30312551

[10] Cutting GR. Cystic fibrosis genetics: from molecular understanding to clinical application. Nat Rev Genet. 2015;16(1):45–56. Epub 2014/11/19. 10.1038/nrg3849 nrg3849 [pii]. ; PMCID: PMC4364438. 25404111 PMC4364438

[11] O'Sullivan BP, Freedman SD. Cystic fibrosis. Lancet. 2009;373(9678):1891–904. 10.1016/S0140-6736(09)60327-5 . 19403164

[12] Sathe MN, Freeman AJ. Gastrointestinal, Pancreatic, and Hepatobiliary Manifestations of Cystic Fibrosis. Pediatr Clin North Am. 2016;63(4):679–98. Epub 2016/07/30. 10.1016/j.pcl.2016.04.008 [pii]. . 27469182

[13] De Lisle RC, Borowitz D. The cystic fibrosis intestine. Cold Spring Harb Perspect Med. 2013;3(9):a009753 Epub 2013/06/22. cshperspect.a009753 [pii] 10.1101/cshperspect.a009753 . 23788646 PMC3753720

[14] Borowitz D. CFTR, bicarbonate, and the pathophysiology of cystic fibrosis. Pediatr Pulmonol. 2015;50 Suppl 40:S24–S30. Epub 2015/09/04. 10.1002/ppul.23247 . 26335950

[15] Hoffman LR, Pope CE, Hayden HS, Heltshe S, Levy R, McNamara S, et al Escherichia coli dysbiosis correlates with gastrointestinal dysfunction in children with cystic fibrosis. Clin Infect Dis. 2014;58(3):396–9. Epub 2013/11/02. cit715 [pii] 10.1093/cid/cit715 ; PMCID: PMC3890337. 24178246 PMC3890337

[16] Matamouros S, Hayden HS, Hager KR, Brittnacher MJ, Lachance K, Weiss EJ, et al Adaptation of commensal proliferating Escherichia coli to the intestinal tract of young children with cystic fibrosis. Proc Natl Acad Sci U S A. 2018;115(7):1605–10. Epub 2018/01/31. 10.1073/pnas.1714373115 ; PMCID: PMC5816161. 29378945 PMC5816161

[17] Manor O, Levy R, Pope CE, Hayden HS, Brittnacher MJ, Carr R, et al Metagenomic evidence for taxonomic dysbiosis and functional imbalance in the gastrointestinal tracts of children with cystic fibrosis. Sci Rep. 2016;6:22493 10.1038/srep22493 ; PMCID: PMC4778032. 26940651 PMC4778032

[18] Dhaliwal J, Leach S, Katz T, Nahidi L, Pang T, Lee JM, et al Intestinal inflammation and impact on growth in children with cystic fibrosis. J Pediatr Gastroenterol Nutr. 2015;60(4):521–6. 10.1097/MPG.0000000000000683 . 25539196

[19] Sutherland R, Katz T, Liu V, Quintano J, Brunner R, Tong CW, et al Dietary intake of energy-dense, nutrient-poor and nutrient-dense food sources in children with cystic fibrosis. J Cyst Fibros. 2018;17(6):804–10. Epub 2018/05/05. 10.1016/j.jcf.2018.03.011 . 29724576

[20] Garg M, Ooi CY. The Enigmatic Gut in Cystic Fibrosis: Linking Inflammation, Dysbiosis, and the Increased Risk of Malignancy. Curr Gastroenterol Rep. 2017;19(2):6 Epub 2017/02/06. 10.1007/s11894-017-0546-0 [pii]. . 28155088

[21] Yamada A, Komaki Y, Komaki F, Micic D, Zullow S, Sakuraba A. Risk of gastrointestinal cancers in patients with cystic fibrosis: a systematic review and meta-analysis. Lancet Oncol. 2018;19(6):758–67. Epub 2018/05/01. S1470-2045(18)30188-8 [pii] 10.1016/S1470-2045(18)30188-8 . 29706374

[22] Chen J, Pitmon E, Wang K. Microbiome, inflammation and colorectal cancer. Semin Immunol. 2017;32:43–53. Epub 2017/10/07. S1044-5323(17)30024-6 [pii] 10.1016/j.smim.2017.09.006 . 28982615

[23] Snouwaert JN, Brigman KK, Latour AM, Malouf NN, Boucher RC, Smithies O, et al An animal model for cystic fibrosis made by gene targeting. Science. 1992;257(5073):1083–8. 10.1126/science.257.5073.1083 . 1380723

[24] Hodges CA, Grady BR, Mishra K, Cotton CU, Drumm ML. Cystic fibrosis growth retardation is not correlated with loss of Cftr in the intestinal epithelium. Am J Physiol Gastrointest Liver Physiol. 2011;301(3):G528–36. Epub 2011/06/11. ajpgi.00052.2011 [pii] 10.1152/ajpgi.00052.2011 ; PMCID: PMC3174541. 21659619 PMC3174541

[25] Leung DH, Heltshe SL, Borowitz D, Gelfond D, Kloster M, Heubi JE, et al Effects of Diagnosis by Newborn Screening for Cystic Fibrosis on Weight and Length in the First Year of Life. JAMA Pediatr. 2017;171(6):546–54. Epub 2017/04/25. 10.1001/jamapediatrics.2017.0206 2617992 [pii]. ; PMCID: PMC5731827. 28437538 PMC5731827

[26] Hardin DS. GH improves growth and clinical status in children with cystic fibrosis—a review of published studies. Eur J Endocrinol. 2004;151 Suppl 1:S81–5. Epub 2004/09/02. 10.1530/eje.0.151s081 . 15339250

[27] Lavelle GM, White MM, Browne N, McElvaney NG, Reeves EP. Animal Models of Cystic Fibrosis Pathology: Phenotypic Parallels and Divergences. Biomed Res Int. 2016;2016:5258727 Epub 2016/06/25. 10.1155/2016/5258727 ; PMCID: PMC4908263. 27340661 PMC4908263

[28] Paik J, Pershutkina O, Meeker S, Yi JJ, Dowling S, Hsu C, et al Potential for using a hermetically-sealed, positive-pressured isocage system for studies involving germ-free mice outside a flexible-film isolator. Gut Microbes. 2015;6(4):255–65. 10.1080/19490976.2015.1064576 ; PMCID: PMC4615381. 26177210 PMC4615381

[29] Clarke LL, Gawenis LR, Bradford EM, Judd LM, Boyle KT, Simpson JE, et al Abnormal Paneth cell granule dissolution and compromised resistance to bacterial colonization in the intestine of CF mice. Am J Physiol Gastrointest Liver Physiol. 2004;286(6):G1050–8. Epub 2004/01/13. 10.1152/ajpgi.00393.2003 00393.2003 [pii]. . 14715526

[30] Nielsen S, Needham B, Leach ST, Day AS, Jaffe A, Thomas T, et al Disrupted progression of the intestinal microbiota with age in children with cystic fibrosis. Sci Rep. 2016;6:24857 Epub 2016/05/05. 10.1038/srep24857 ; PMCID: PMC4855157. 27143104 PMC4855157

[31] Mulcahy EM, Hudson JB, Beggs SA, Reid DW, Roddam LF, Cooley MA. High peripheral blood th17 percent associated with poor lung function in cystic fibrosis. PLoS One. 2015;10(3):e0120912 10.1371/journal.pone.0120912 ; PMCID: PMC4372584. 25803862 PMC4372584

[32] Tiringer K, Treis A, Fucik P, Gona M, Gruber S, Renner S, et al A Th17- and Th2-skewed cytokine profile in cystic fibrosis lungs represents a potential risk factor for Pseudomonas aeruginosa infection. Am J Respir Crit Care Med. 2013;187(6):621–9. Epub 2013/01/12. 10.1164/rccm.201206-1150OC rccm.201206-1150OC [pii]. . 23306544

[33] Safe M, Gifford AJ, Jaffe A, Ooi CY. Resolution of Intestinal Histopathology Changes in Cystic Fibrosis after Treatment with Ivacaftor. Ann Am Thorac Soc. 2016;13(2):297–8. 10.1513/AnnalsATS.201510-669LE . 26848606

[34] Andersen DH. Cystic fibrosis of the pancreas and its relation to celiac disease—A clinical and pathologic study. Am J Dis Child. 1938;56(2):344–99. 10.1001/archpedi.1938.01980140114013 ISI:000201769200013.

[35] Vaishnava S, Behrendt CL, Ismail AS, Eckmann L, Hooper LV. Paneth cells directly sense gut commensals and maintain homeostasis at the intestinal host-microbial interface. Proc Natl Acad Sci U S A. 2008;105(52):20858–63. Epub 2008/12/17. 0808723105 [pii] 10.1073/pnas.0808723105 ; PMCID: PMC2603261. 19075245 PMC2603261

[36] Vaishnava S, Yamamoto M, Severson KM, Ruhn KA, Yu X, Koren O, et al The antibacterial lectin RegIIIgamma promotes the spatial segregation of microbiota and host in the intestine. Science. 2011;334(6053):255–8. Epub 2011/10/15. 334/6053/255 [pii] 10.1126/science.1209791 ; PMCID: PMC3321924. 21998396 PMC3321924

[37] Clevers HC, Bevins CL. Paneth cells: maestros of the small intestinal crypts. Annu Rev Physiol. 2013;75:289–311. 10.1146/annurev-physiol-030212-183744 . 23398152

[38] Flass T, Tong S, Frank DN, Wagner BD, Robertson CE, Kotter CV, et al Intestinal lesions are associated with altered intestinal microbiome and are more frequent in children and young adults with cystic fibrosis and cirrhosis. PLoS One. 2015;10(2):e0116967 10.1371/journal.pone.0116967 ; PMCID: PMC4319904. 25658710 PMC4319904

[39] van Elburg RM, Uil JJ, van Aalderen WM, Mulder CJ, Heymans HS. Intestinal permeability in exocrine pancreatic insufficiency due to cystic fibrosis or chronic pancreatitis. Pediatr Res. 1996;39(6):985–91. 10.1203/00006450-199606000-00010 . 8725259

[40] Kushwah R, Gagnon S, Sweezey NB. Intrinsic predisposition of naive cystic fibrosis T cells to differentiate towards a Th17 phenotype. Respir Res. 2013;14:138 Epub 2013/12/19. 10.1186/1465-9921-14-138 1465-9921-14-138 [pii]. ; PMCID: PMC3890528. 24344776 PMC3890528

[41] Moss RB, Hsu YP, Olds L. Cytokine dysregulation in activated cystic fibrosis (CF) peripheral lymphocytes. Clin Exp Immunol. 2000;120(3):518–25. Epub 2000/06/09. cei1232 [pii] 10.1046/j.1365-2249.2000.01232.x ; PMCID: PMC1905557. 10844532 PMC1905557

[42] Brazova J, Sediva A, Pospisilova D, Vavrova V, Pohunek P, Macek M Jr., et al Differential cytokine profile in children with cystic fibrosis. Clin Immunol. 2005;115(2):210–5. Epub 2005/05/12. S1521-6616(05)00037-9 [pii] 10.1016/j.clim.2005.01.013 . 15885645

[43] Ooi CY, Syed SA, Rossi L, Garg M, Needham B, Avolio J, et al Impact of CFTR modulation with Ivacaftor on Gut Microbiota and Intestinal Inflammation. Sci Rep. 2018;8(1):17834 Epub 2018/12/14. 10.1038/s41598-018-36364-6 ; PMCID: PMC6292911. 30546102 PMC6292911

[44] Klindworth A, Pruesse E, Schweer T, Peplies J, Quast C, Horn M, et al Evaluation of general 16S ribosomal RNA gene PCR primers for classical and next-generation sequencing-based diversity studies. Nucleic Acids Res. 2013;41(1):e1 Epub 2012/08/31. 10.1093/nar/gks808 gks808 [pii]. ; PMCID: PMC3592464. 22933715 PMC3592464

[45] Martin M. Cutadapt removes adapter sequences from high-throughput sequencing reads. 2011. 2011;17(1):3 Epub 2011-08-02. 10.14806/ej.17.1.200

[46] Callahan BJ, McMurdie PJ, Rosen MJ, Han AW, Johnson AJ, Holmes SP. DADA2: High-resolution sample inference from Illumina amplicon data. Nat Methods. 2016;13(7):581–3. Epub 2016/05/24. 10.1038/nmeth.3869 nmeth.3869 [pii]. ; PMCID: PMC4927377. 27214047 PMC4927377

[47] McMurdie PJ, Holmes S. phyloseq: an R package for reproducible interactive analysis and graphics of microbiome census data. PLoS One. 2013;8(4):e61217 Epub 2013/05/01. 10.1371/journal.pone.0061217 PONE-D-12-31789 [pii]. ; PMCID: PMC3632530. 23630581 PMC3632530

[48] Parks DH, Tyson GW, Hugenholtz P, Beiko RG. STAMP: statistical analysis of taxonomic and functional profiles. Bioinformatics. 2014;30(21):3123–4. Epub 2014/07/26. 10.1093/bioinformatics/btu494 ; PMCID: PMC4609014. 25061070 PMC4609014

[49] Benjamini Y, Hochberg Y. Controlling the False Discovery Rate: A Practical and Powerful Approach to Multiple Testing. Journal of the Royal Statistical Society Series B (Methodological). 1995;57(1):289–300. citeulike-article-id:1042553 10.2307/2346101

