# Supplementary figures and images for: CFTR dysregulation drives active selection of the gut microbiome
Source: PLoS Pathog. 2020 Jan 21;16(1):e1008251. doi: 10.1371/journal.ppat.1008251 (PMC6994172; doi:10.1371/journal.ppat.1008251)

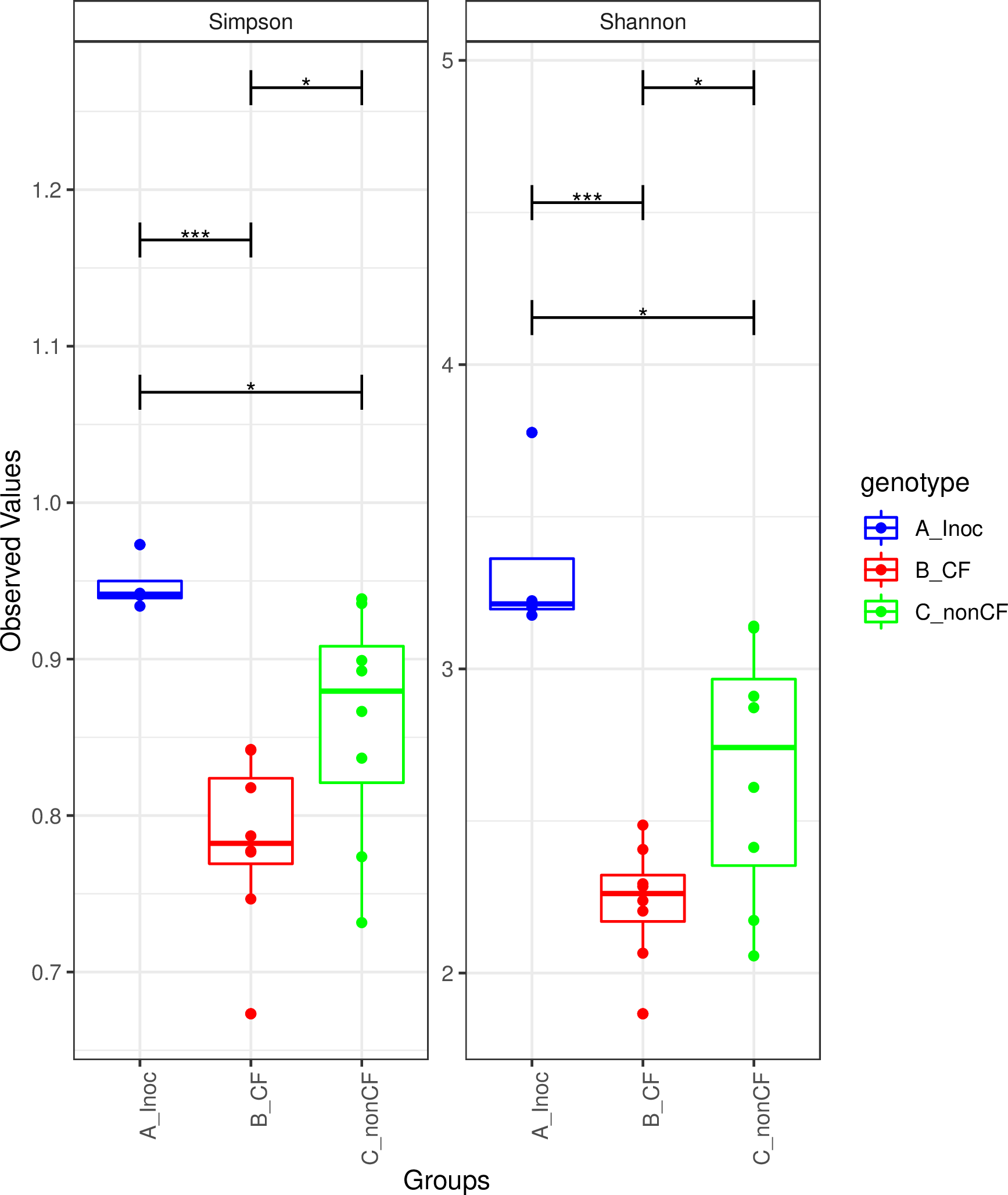

Supplement: S1 Fig — Alpha-diversity, measured by Simpson and Shannon diversity indexes is plotted for fecal and cecal microbiome samples assessed in CF (red) and non-CF (green) germ-free recipient mice 1–3 months following FMT with SPF C57BL/6J feces as the inoculum (blue). Fecal and cecal contents were pooled at equal volumes for pair-housed, age, sex, and genotype matched mice within a study prior to DNA extraction (N = 2 mice per genotype per experiment, four separate experiments performed).The line inside the box represents the median, while the whiskers represent the lowest and highest values within the 1.5 interquartile range (IQR). Pair-wise ANOVA of diversity measures was performed between groups and values for each of the selected methods (observed values) were plotted (annotated with significance labels:*p<0.05; **p<0.01; ***p<0.001). (TIF) [file ppat.1008251.s001.tif]

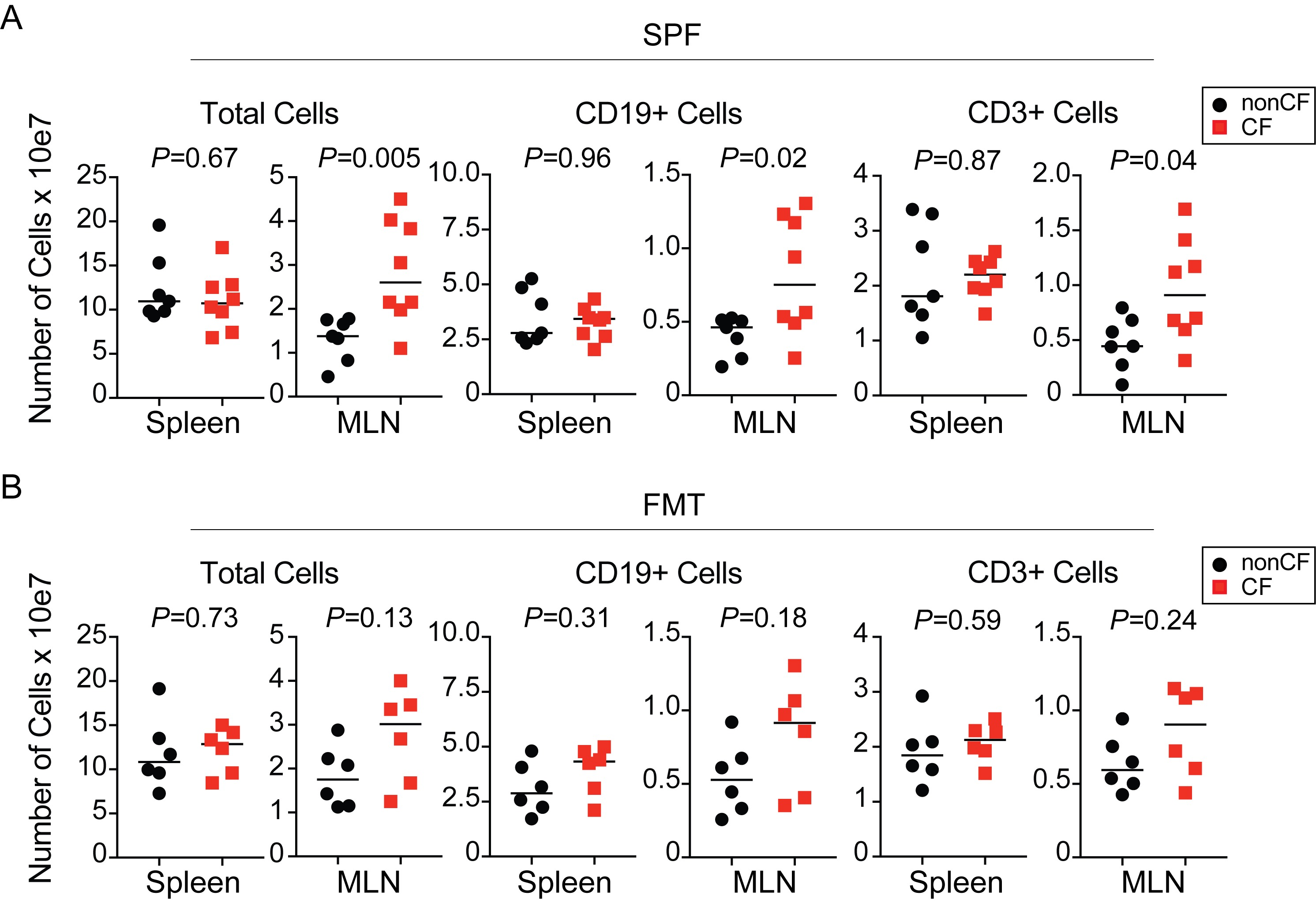

Supplement: S2 Fig — Total cell counts in addition to number of B cells (CD19+) and T cells (CD3+) present in Spleen and MLN from CF and non-CF mice in SPF [(A) N = 7–8 mice per genotype] or B6-FMT mice [(B) N = 6 mice per genotype, not performed in 1st FMT experiment]. Data were analyzed by pairwise comparison between CF and non-CF animals within a group by Mann-Whitney U test. (TIF) [file ppat.1008251.s002.tif]
