# Supplementary material for: CFTR dysregulation drives active selection of the gut microbiome
Source: PLoS Pathog. 2020 Jan 21;16(1):e1008251. doi: 10.1371/journal.ppat.1008251 (PMC6994172; doi:10.1371/journal.ppat.1008251)
Supplement: S1 Table — (DOCX) [file ppat.1008251.s003.docx]

| **ANTIBODIES and REAGENTS** | **SOURCE** | **CATALOG NUMBER** |
| --- | --- | --- |
| APC anti-mouse CD8a | Biolegend | Cat# 100712 |
| Alexa Fluor 700 anti-mouse CD4 | Biolegend | Cat# 100536 |
| APC-Cy7 rat anti-mouse CD19 | BD Biosciences | Cat# 557655 |
| Pacific Blue anti-mouse CD3ε | Biolegend | Cat# 100334 |
| PE anti-mouse CD8a | Biolegend | Cat# 100708 |
| PE anti-mouse IFN-γ | BD Biosciences | Cat# 554412 |
| PE/Cy7 anti-mouse CD8a | Biolegend | Cat# 100722 |
| FITC anti-mouse CD8a | Biolegend | Cat# 100706 |
| FITC anti-mouse IL-17A | Biolegend | Cat# 506908 |
| PerCP/Cyanine5.5 anti-mouse CD4 | Biolegend | Cat# 100540 |
| Rat IgG1 FITC | Biolegend | Cat# 400406 |
| Rat IgG1 PE | BD Biosciences | Cat# 553925 |
| Rat IgG2b APC | Biolegend | Cat# 400612 |
| Rat IgG1 Alexa Fluor 700 | Biolegend | Cat# 400420 |
| Mouse IgG2a PerCP Cy5.5 | BD Biosciences | Cat# 550927 |
| BD Cytofix/Cytoperm Buffer | BD Biosciences | Cat# 51-2090KZ |
| BD Perm/Wash Buffer | BD Biosciences | Cat# 51-2091KZ |
